# Physician Assistants and Nurse Practitioners in Primary Care Plus: A Systematic Review

**DOI:** 10.5334/ijic.5485

**Published:** 2021-02-12

**Authors:** R. M. A. van Erp, A. L. van Doorn, G. T. van den Brink, J. W. B. Peters, M. G. H. Laurant, A. J. van Vught

**Affiliations:** 1 HAN University of Applied Sciences, Verlengde Groenestraat 75, 6525 EJ, Nijmegen, the Netherlands; 2 Master Advanced Nursing Practice, HAN University of Applied Sciences, Groenewoudseweg 1, 6524 TM, Nijmegen, the Netherlands; 3 HAN University of Applied Sciences, Faculty of Health and Social Studies, Kapittelweg 33, 6525 EN, Nijmegen, the Netherlands

**Keywords:** physician assistants, nurse practitioners, primary health care, systematic review, substitution of care, integrated care

## Abstract

**Introduction:**

Shifting specialist care from the hospital to primary care/community care (also called primary care plus) is proposed as one option to reduce the increasing healthcare costs, improve quality of care and accessibility. The aim of this systematic review was to get insight in primary care plus provided by physician assistants or nurse practitioners.

**Methods:**

Scientific databases and reference list were searched. Hits were screened on title/abstract and full text. Studies published between 1990–2018 with any study design were included. Risk of bias assessment was performed using QualSyst tool.

**Results:**

Search resulted in 5.848 hits, 15 studies were included. Studies investigated nurse practitioners only. Primary care plus was at least equally effective as hospital care (patient-related outcomes). The number of admission/referral rates was significantly reduced in favor of primary care plus. Barriers to implement primary care plus included obtaining equipment, structural funding, direct access to patient-data. Facilitators included multidisciplinary collaboration, medical specialist support, protocols.

**Conclusions and Discussion:**

Quality of care within primary care plus delivered by nurse practitioners appears to be guaranteed, at patient-level and professional-level, with better access to healthcare and fewer referrals to hospital. Most studies were of restricted methodological quality. Findings should be interpreted with caution.

## Introduction

While life expectancy of people living in Europe increases rapidly [1], the number of people with multiple chronic diseases (multimorbidity) increases along. One-third of the people aged 55 years and older experiences multimorbidity according to a Dutch general practitioner database [2]. The proportion of people of ≤65 years is expected to increase from 14% (2010) to 25% (2050) in the European region [1]. Therefore, the number of people with multimorbidity is expected to increase in the near future as well.

Patients with multimorbidity require specialist health care, which is usually provided in a hospital or a specialized clinic. Specialist care in the hospital setting can however be very expensive. In combination with the expected increase in patients with multimorbidity, Western countries face the need to change the health care system to control the increasing health care costs [3]. One option is integrating specialist care from the hospital setting to the primary care setting or community care setting; in other words, care provided at patients’ home or close to patients’ home.

In the past years many studies have been performed about integrated models of care provision, for example extensive care, transmural care and collaborative care. The umbrella term for these models of care is integrated care. The aim of integrated care is achieving more care beyond the hospital walls, change in the size and shape of acute hospitals, and increased attention to prevention and population health [4]. Also primary care plus is a model of integrated care. In primary care plus, specialist care which was previously performed by a medical specialist in a hospital or (outpatient) clinic is now provided in primary care or community care, close to the patients’ home [5]. This model is roughly equivalent to a patient-centred medical home programme as are common in the US, which provides comprehensive, coordinated and continuous primary care close to patient’s home [6].

Primary care plus was developed with the aim of creating substitution and stimulating integrated care by allowing medical specialists to perform consultations within primary care. For example, a cardiologist providing a consultation in a general practitioner practice [7] which was previously provided in the hospital setting. In contrast to integrated care, primary care plus only focuses on substitution of specialist medical care usually performed by medical specialists from hospital to primary health care. Primary care plus has two goals; either preventing patients to be referred to a hospital (specialized screening and treatment), or earlier hospital discharge (specialized treatment). A potential advantage of primary care plus for patients is the prevention from hospitalization (and possibly over diagnosing), early discharge, and health care delivery close to or at patient’s homes [8,9].

A recent systematic review of van Hoof et al. (2019) investigated the difference in effectiveness between specialist hospital care and primary care plus. Included initiatives were located in the UK (n = 10), the Netherlands (n = 3) and Spain (n = 1) [10]. They reported at least equal effectiveness, shorter waiting lists/times and higher patient satisfaction in favour of primary care plus. In these initiatives, specialist care was mainly provided by a medical specialist, whether or not in collaboration with a general practitioner. The question is whether other health care professionals such as a physician assistant or nurse practitioner could play a role in primary care plus.

Physician assistants and nurse practitioners both work at a Master degree level and are trained to take over medical tasks independently from doctors [11,12]. Physician assistants work in the medical domain, which means that they provide patient consultations and visits (direct-patient care), but also develop or improve protocols and provide training to clinical colleagues (indirect patient care). Nurse practitioners, on the other hand, work in both the medical and the nursing domain. They primarily focus on specific diseases and become experts in that field. In the Netherlands, physician assistants and nurse practitioners work independently and are authorized to perform specified reserved medical procedures [13,14].

Previous systematic reviews have shown that both professionals can effectively and safely provide tasks and responsibilities, which were usually performed by medical specialists [15,16,17]. To what extend substitution of specialist care by a physician assistant or nurse practitioner is possible or effective within primary care plus is less investigated. No systematic review has yet been performed which investigated the delivery of care by both professionals in primary care plus.

### Research Aim

The aim of this systematic review was to provide an overview of studies evaluating primary care plus services provided by a physician assistant and/or nurse practitioner in a team of health care professionals. In particularly, we were interested in the roles of both professionals within primary care plus, the effectiveness (at patient and professional level), costs and influencing factors (barriers and facilitators).

## Methods

### Design

A systematic review was performed according to the Cochrane Collaboration guidelines. Furthermore, it was registered in the International Register of Systematic Reviews (PROSPERO; available from *https://www.crd.york.ac.uk/PROSPERO/*; registration number: CRD42018088423; 12 February 2018).

### Eligibility Criteria

Original national and international studies with any study design (either qualitative or quantitative), written in English or Dutch and published between January 1990 and 2018 as peer-reviewed article were eligible for the systematic review. Letters, personal stories, editorials, conference abstracts, reviews and meta-analyses were not included in the systematic review.

Studies had to investigate primary care plus which we defined as ‘specialist care which is usually provided by a medical specialist, physician assistant or nurse practitioner in a hospital, but which is now provided (or integrated) in primary care setting or community care’, face-to-face, by a physician assistant or nurse practitioner with specific expertise in this patient population.

Exclusion criteria for primary care plus included: solely “additional care” which is provided in addition to usual care, which has not been provided previously and which aims to increase the quality of care (e.g. heart failure screening or follow-up care after hospital discharge which was not provided before primary care plus was introduced); primary care provided by a physician assistant/nurse practitioner; substituted care from mental health services, nursing homes, hospice or rehabilitation centres to primary care; a nurse-led clinic in a hospital; telephone consultations by a hospital-based physician assistant/nurse practitioner; educational programs provided by a physician assistant/nurse practitioner to improve self-management of patients (e.g. patients learn to perform injections themselves); health care which would normally be provided by a practice nurse in the Dutch health care system (e.g. monitoring of patients with stable chronic diseases, including given advice and education according to evidence based protocols) [12].

Primary care plus had to be provided by a physician assistant and/or nurse practitioner qualified with a master’s degree (EQF 7). Since different synonyms are used for physician assistant and nurse practitioner, studies evaluating a Physician Assistant, Physician Associate, Nurse Specialist, Nurse Practitioner, Clinical Nurse Specialist, Advanced Practice Nurse or Advanced Nurse Practitioner were included. No restrictions were imposed on age, gender, ethnic or other demographic characteristics, or the number of years spent working. In addition, no restrictions were set for the patient population except that primary care plus service had to be provided by a physician assistant and/or nurse practitioner.

Primary outcomes of interest for the systematic review included “patient outcomes” (morbidity, mortality, health status, quality of life, patient satisfaction, patient compliance, referral to hospital, admission, and patient safety), “care outcomes” (health care activities/roles such as examination, advice, treatments; the quality of the health care; and facilitators and barriers), “provider outcomes” (job workload, job satisfaction, and the experiences of physician assistants/nurse practitioners/medical specialists), and “costs and cost-effectiveness” (including utilization of resources).

### Literature Search

A search strategy was developed by multiple authors (RvE, GvdB, ML and AvV) and optimized by an information specialist (T.P.) working at the HAN university of Applied Sciences (HAN). The search strategy included a combination of indexed keywords such as Medical Subject Headings (MeSH) and text terms, which were searched on title/abstract (Appendix 1). Since there is no specific term for “primary care plus service” the search strategy included a broad range of related terms to increase the chance of identifying relevant studies (higher sensitivity, lower specificity). The search strategy included e.g. (Integrated) health care delivery, health care reform, consultation, liaison, hospital based home care, and etcetera.

The information specialist conducted the search in February 2018 and used the following databases: CINAHL (EBSCO), Cochrane Database of Systematic Reviews (CDSR; Cochrane Library: Wiley), Embase (Ovid), PubMed (NLM; Internet, *http://www.ncbi.nlm.nih.gov/pubmed*) and Web of Science. At a later stage (February 2019), reference lists of included articles and (systematic) reviews were screened for additional eligible studies. Search records were downloaded, collected and de-duplicated using EndNote bibliographic software (Clarivate Analytics, Philadelphia, PA, U.S.A.). Afterwards, search records were exported to Rayyan QCRI [18] for the selection procedure.

### Study Selection/Selection Methods

Three review authors (RvE, AvD and AvV) performed the study selection procedure. Records were first sorted on relevance. Title and abstracts of the first 30 records were independently screened by all three review authors. Review authors discussed interpretation of eligibility criteria. Next, all records were sorted alphabetically and the first 1,500 records were independently screened by two reviewers on title/abstract. Screening results were discussed between the two review authors and if necessary, screened by a third review author to resolve disagreement. The remaining records (4,348 records) were divided over the three review authors and screened on title and abstract. Only in case of selection for inclusion and when in doubt for selection for inclusion, a second review author was involved for final inclusion. Next, all papers identified to be included based on title and abstract were full text screened. Again, records were divided over three review authors and screened. In case of selection for inclusion and when in doubt for selection for inclusion a second review author was involved for final decision (i.e. final inclusion of the study in the systematic review). Reference lists of included articles were subsequently screened on relevant articles. In addition, reference lists of relevant review articles derived from the search were screened as well.

### Risk of Bias Assessment

Two review authors (RvE and AvD) performed the risk of bias assessment using the Quality Assessment Tool for Quantitative Studies (QualSyst tool) [19]. The QualSyst tool is developed by the Effective Public Health Practice Project (EPHPP, Canada) for Public Health purposes and can be used for the assessment of studies with varying study designs. It is therefore a suitable instrument to be used in this systematic review. The risk of bias assessment was performed by one review author and checked by another. Kmet et al. (2004) defines studies with a sum score of ≥0.5 of adequate quality. We choose however not to exclude studies based on the sum score. The topic of this systematic review is in its infancy and therefore frequently studied in non-randomised studies and/or reported descriptively. Excluding studies with a low sum score would give a narrowed insight in the roles and tasks from physician assistants and nurse practitioners in primary care plus.

### Data Extraction

One review author (RvE, AvD or AvV) extracted data from the included studies using a predefined data extraction form for quantitative and qualitative data. Another review author (RvE, AvD or AvV) checked the extracted data. Relevant extracted data included i.e. author, publication date, study design, participants, intervention(s), outcomes and results. In addition, and if deemed necessary, the corresponding author of the study was contacted to clarify extracted data. It was not possible (and not planned) to perform a meta-analysis as this systematic review allowed studies with varying research designs, populations, health care settings, interventions and outcomes.

## Results

**Figure 1:**
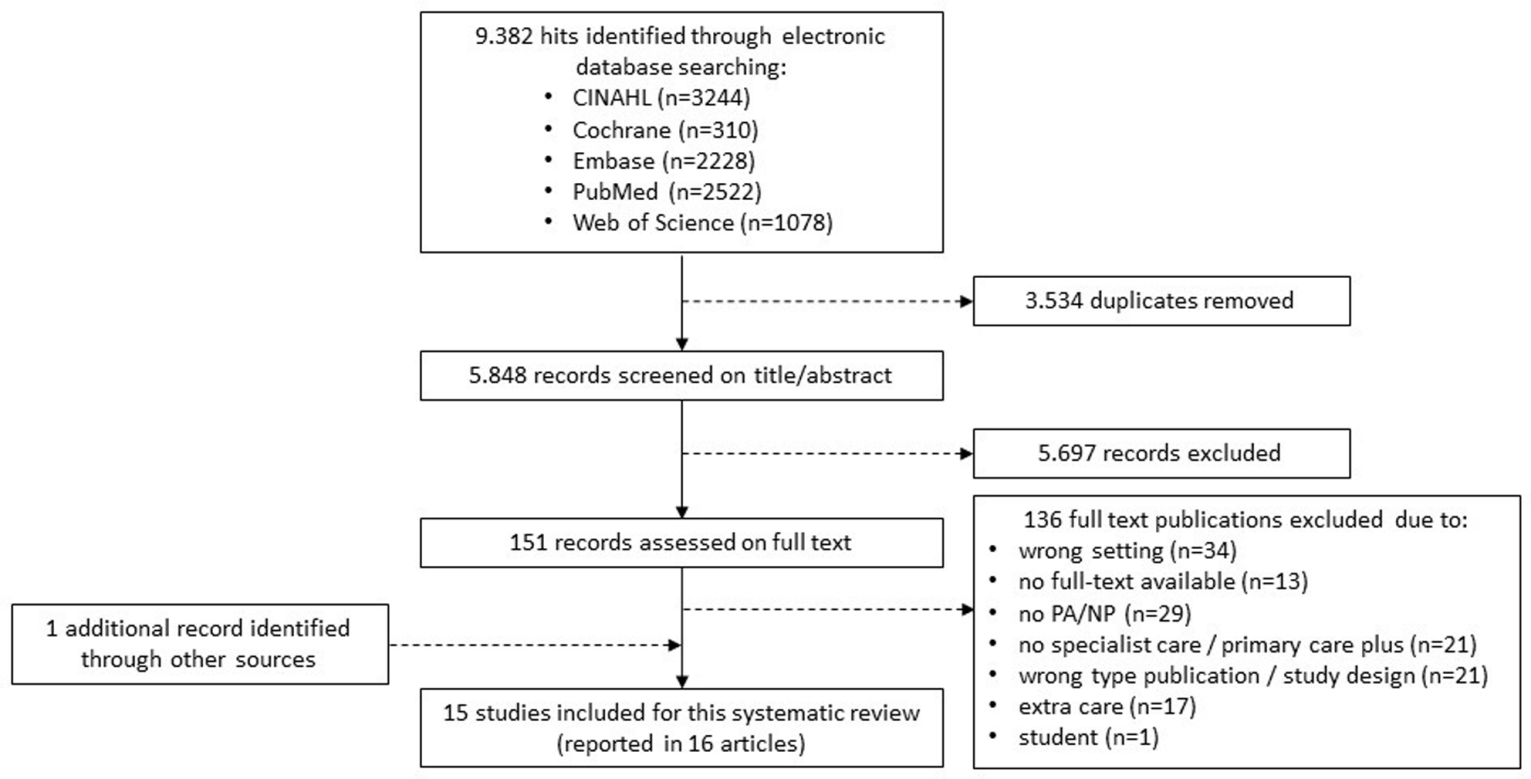
Study flow diagram. PA = physician assistant, NP = nurse practitioner.

The search resulted in 9,382 hits (***Figure 1***). After de-duplicating, 5,848 hits remained and were screened on title and abstract. Of these, 152 hits were screened on full text. One additional hit was identified by checking reference lists of relevant systematic reviews. Eventually 15 studies, reported in 16 articles, met the inclusion criteria and were therefore included in the systematic review.

### Characteristics of Included Studies

Included studies were performed in USA (n = 5), Canada (n = 1), New Zealand (n = 2) and the United Kingdom (n = 7). Study designs ranged from randomized controlled trials (RCT; n = 3), pre-post single patient group designs (n = 3), cohort studies (n = 2), to observational descriptive studies (n = 7). Publication date ranged between 2000 and 2016.

### Participants

All included studies investigated the implementation of a nurse practitioner in primary care plus (***Table 1***). No studies investigated the implementation of a physician assistant. Some studies used other synonyms for nurse practitioner (e.g. (clinical) nurse specialist, advanced practice nurse or advanced nurse practitioner). Since these professions all require a Master degree [11,12], they are collectively mentioned as nurse practitioner in this systematic review. The number of nurse practitioners involved in the studies varied from one nurse practitioner to a team of nurse practitioners. Most studies lacked information about the characteristics of the nurse practitioners such as education level, years of working experience and the degree of autonomy. If they did report this, years of experience varied from 5 [8] to 25 years [20]. More than half of the studies reported supervision by medical specialists (e.g. family medicine physician, cardiologist, radiologist, clinical doctor) [8,21,22,23,24,25,26,27,28]. Supervision occurred at weekly meetings or in case of complications. In addition to face-to-face consultations, two studies reported support by virtual technology (including webinars and telehealth), software (drawing tool and administration system), and/or decision-making tools (pocket-cards and summarized guideline templates) [29,30]. Others did not report supervision nor supportive tools [9,20,31,32].

**Table 1:** Characteristics of the included studies.


STUDY	COUNTRY	DESIGN	PARTICIPANTS	EDUCATION/RIGHTS	SPECIALIZATION	PRIMARY CARE PLUS	CONTROL INTERVENTION	AI

Ansari et al. (2009)	UK	Observational cohort study	NP (a team)	N.R.	COPD	Urgent Care Team (a team of NPs) provides ‘hospital-at-home’ to patients with an acute exacerbation of COPD. NP treats patients with nebulized bronchodilators, prednisolone and doxycycline. Support: N.R.	Usual hospital care	Preventing referral

Bookbinder et al. (2011)	USA	Descriptive study	NP (n = 1)	Advanced training in palliative care	Palliative care	Palliative home care team (secondary care NP and social worker) provides consultation and direct home care for homebound elderly with advanced illnesses. Support: Department of Pain Medicine and Palliative Care (nursing, social work, and medicine).	N.A.	Preventing referral

Gunn et al. (2000)	New Zealand	RCT	NS (a team)	Specially skilled nurses with neonatal nursing experience	Preterm infants	Team of NSs provides home support for preterm infants (daily home care visits for 7–10 days after early discharge, including weekends), and are 24h a day availably by telephone. Support: N.R.	Routine care; hospital care and daily standard home care including home visits/telephone contact by home care nurses for 5 weekdays after discharge.	Early discharge

Jack et al. (2008)	UK	Descriptive study	CNS (n = 1)	CNS does not prescribe opiate substitutes	Hepatitis C virus (HCV)	CNS in hepatitis forms a partnership with drug workers and GPs in a general practice. The CNS provides HCV consultations (screening, diagnosing and treatment). Supervision: secondary-care-based consultant in infectious diseases.	N.A.	Preventing referral

Kemp, A.E. (2016)	UK	Observational study	ANP (n = 1)	An independent prescriber and accredited prehospital care practitioner; have master’s level post-registration qualifications.	First aid	ANP works alongside an event medical team (paramedics and first aiders) at a mass-gathering event. ANP assesses, diagnoses, and provides treatments and advice (e.g. wound closures, prescribe medication). Support: N.R.	Usual care provided by the event medical team (without ANP).	Preventing referral

Lemelin et al. (2007)	Canada	Descriptive study	NP (n = 6)	Licensed as extended class RN’s; educated as Primary Care NP’s	Family Medicine	NPs provide daily home visits and telephone contact. The NPs performs physical examination and initiates care provision; rehabilitative and supportive care, including education, coordination of services, and counseling. Support and supervision: Family Medicine physicians.	N.A.	Preventing referral

Lucatorto et al. (2016)	USA	Pilot study, Pre–post, single-patient group design	NP (n = 1)	Nurses with advanced training and scopes of practice that include diagnosing disease and prescribing treatment	Diabetes and chronic kidney disease	Advanced Practice Registered Nurse (APRN)-Led Specialty Care Team for patients with diabetes and chronic kidney disease. The APRN-team consists of a NP, RN, licensed PN, RN certified diabetes educator, registered dietitian and clinical pharmacist. NP provides clinical examination and medication adjustment, is responsible for communicating the team plan, treatment changes, and summary of care to the primary care provider. Support: virtual technology and clinical decision making tools.	N.A.	Preventing referral

Lukas et al. (2013)	USA	Pre–post, single-group design	NP (n = 3)	N.R.	Palliative care	Physicians and NPs within a palliative medicine practice for elderly with advanced complex illness. Outpatient arm provides home-based, non-hospice palliative medicine consultation and management. NPs deliver direct care, e.g. symptom management, advanced care planning, goal-directed care and care coordination. Support: collaborating physician (20%).	Usual care (situation prior to the introduction of the palliative medicine practice)	Preventing referral

Maruthachalam et al. (2006)	UK	Observational study	NS (n = 1)	N.R.	Flexible sigmoidoscopy	Flexible sigmoidoscopy clinic developed at GP practice. Secondary care personnel delivers care; nurse endoscopist, colorectal nurse specialist, endoscopy nurse and auxiliary nurse. The colorectal NS manages benign conditions (e.g. haemorrhoids and anal fissures), provides verbal and written advice, books follow-up appointments. Support: protocols, contact with physician, weekly meetings to review patients (lead consultant and nurse endoscopist).	Secondary care	Preventing referral

McCorkle et al. (2000)	USA	RCT	APN (multiple)	Masters-prepared clinicians	Post-surgical cancer	APNs deliver 4-week specialized home care to post-surgical older patients with cancer (3 home visits, 5 telephone contacts). APNs provide education (43%), assessment and monitoring health status (25%), psychological support (16%), referrals (11%), others (5%). APNs are 24/7 available. Support: standardized protocol, physicians.	Usual post-operative hospital care and routine follow-up care in an ambulatory setting	Early discharge

McLachlan et al. (2015)	New Zealand	Cohort study (descriptive)	NP (n = 1)	NPs can practice autonomously or as part of team, and have prescribing rights	Post-surgical heart valve	NP-led clinic in community-based ambulatory care setting for patients following valve repair/replacement who require long-term follow-up. NP assesses patients annually/biannually, prepares review letter. Support and supervision: senior cardiologist (patient reviewing).	N.A.	Preventing referral

Moore, J.A. (2016)	USA	Pre-post, single-group design	NP (n = 2)	Depending on state: license for prescribing rights and full practice authority	Congestive heart failure	NP-led home-based clinic pathway for patients with congestive heart failure (5 days/week home visits, tele monitoring and weekly telephone contacts). Interdisciplinary team is involved including NPs, RNs, physiotherapists, occupational therapists, a dietician, pharmacists, social workers, and home health aides. NP provides history assessments, physical assessments, education, reconciles medication, reviews clinical pathway and CHF self-management tool, teaches and reviews tele monitoring equipment, and reviews tele monitoring data and follow-up. Support is not reported.	Usual care (situation prior to the introduction of the clinic pathway)	Preventing referral

Regan & Morgan (2015)	UK	Descriptive study	ANP (n = 2)	N.R.	Intravenous antibiotic service	Community-based service by two ANPs and core district nursing service for patients requiring IV antibiotics. ANPs assess patients, visits patients at home, functions as clinical leads and coordinators, promote the service in secondary care, and provide training and support for the district nursing team. Consultant in secondary care keeps final responsibility for patients. Support: radiologist, microbiologists and pharmacists.	N.A.	Early discharge

Tozer & Lugton (2007)	UK	Descriptive study	NS (n = 2)	N.R.	Genetic cancer screening	Nurse-led community service for people concerned about cancer. NS assesses the level of familial cancer risk, triages and refers patients, writes personalized letters, and provides advice. Support: innovative software.	N.A.	Preventing referral

Whitaker et al. (2001)	UK	RCT	NP (n = 1)	N.R.	Botulinum toxin injection for Dystonia	NP provides botulinum toxin injections at home. NP is allowed to make external medical, nursing, or therapy referrals. Support: clinical doctors.	Usual care (injections provided by medical staff in the outpatient clinic)	Preventing referral


NP = nurse practitioner; COPD = Chronic Obstructive Pulmonary Disease; N.R. = not reported; N.A. = not applicable; NS = nurse specialist; RCT = Randomized Controlled Trial; CNS = clinical nurse specialist; ANP = advanced nurse practitioner; APN = advance practice nurse; RN = registered nurse; PN = practice nurse.

### Interventions

As described in the Introduction, primary care plus can be classified into two groups based on the goal of the intervention: (1) preventing referral to a hospital, or (2) stimulating early discharge from the hospital to the home situation. Most of the included studies, concentrated on the first goal (n = 12). Care was usually regular hospital care integrated in primary care whether or not in combination with extra follow-up assessments. If primary care plus was compared with a control intervention, the control intervention consisted of usual specialist hospital care (***Table 1***). Primary care plus was mostly developed for patients with chronic and/or well-defined health issues, e.g. Chronic Obstructive Pulmonary Disease, hepatitis C virus, diabetes, kidney disease, lower gastrointestinal tract symptoms, heart disease, cancer, dystonia, and patients requiring IV antibiotics. Only few primary care plus interventions was developed for general illnesses/health care, e.g. palliative care for elderly with advanced illnesses, preterm infants, first aid, and family medicine.

### Risk of Bias Assessment

The risk of bias assessment resulted in a sum score ranging from 0.19 [27,30] to 0.89 [28] (***Table 2***). Despite low sum scores, no studies were excluded from the analysis as mentioned in the methods section. As became clear, random allocation and blinding of investigators and subjects was not applicable in 12/15 included studies. Furthermore, the sample size was appropriate in two studies only [28,31], and 4/15 studies controlled for confounding [9,23,25,28]. Studies having a very low sum score [27,30] both used a descriptive design in which the selection and characteristics of participants were insufficiently described, and results were not reported in sufficient detail.

**Table 2:** Risk of bias assessment.


STUDIES	QUESTION	STUDY DESIGN	SELECTION	SUBJECT CHARACTERISTICS	RANDOM ALLOCATION	BLINDING INVESTIGATORS	BLINDING SUBJECTS	OUTCOME	SAMPLE SIZE	ANALYTIC METHODS	ESTIMATE OF VARIANCE	CONFOUNDING	RESULTS	CONCLUSION	SUMMARY SCORE*

Ansari et al. (2009)	2	1	1	2	n/a	n/a	n/a	2	2	2	2	0	2	2	18/22 = 0.82

Bookbinder et al. (2011)	1	1	1	2	n/a	n/a	n/a	2	1	2	2	1	2	2	17/22 = 0.77

Gunn et al. (2000)	2	2	2	2	2	n/a	n/a	2	1	2	0	2	2	2	21/24 = 0.88

Jack et al. (2008)	1	1	1	1	n/a	n/a	n/a	1	n/a	n/a	n/a	n/a	1	0	6/14 = 0.43

Kemp, A.E. (2016)	2	2	2	1	n/a	n/a	n/a	2	1	2	n/a	0	2	1	15/20 = 0.75

Lemelin, J. et al. (2007)	2	2	1	2	n/a	n/a	n/a	1	n/a	n/a	n/a	n/a	2	1	11/14 = 0.79

Lucatorto et al. (2016)	0	1	1	1	n/a	n/a	n/a	1	n/a	n/a	n/a	n/a	1	0	5/14 = 0.36

Lukas et al. (2013)	2	2	2	2	n/a	n/a	n/a	2	1	2	1	2	2	1	19/22 = 0.86

Maruthachalam et al. (2006)	1	2	2	1	n/a	n/a	n/a	1	1	n/a	n/a	n/a	2	1	11/16 = 0.69

McCorkle et al. (2000)	2	2	2	2	2	0	0	2	1	2	2	2	2	2	23/28 = 0.82

McLachlan et al. (2015)	2	2	1	2	n/a	n/a	n/a	2	n/a	2	2	0	2	2	17/20 = 0.85

Moore, J.A. (2016)	2	1	1	1	n/a	n/a	n/a	2	0	1	2	0	2	2	14/22 = 0.64

Regan & Morgan (2015)	1	1	0	0	n/a	n/a	n/a	0	0	n/a	n/a	n/a	0	2	3/16 = 0.19

Tozer & Lugton (2007)	1	1	0	0	n/a	n/a	n/a	n/a	n/a	0	0	n/a	0	1	3/16 = 0.19

Whitaker et al. (2001)	2	2	2	2	2	2	0	1	2	2	2	2	2	2	25/28 = 0.89


* Total sum = (number of “yes” * 2) + (number of “partials” * 1); total possible sum = 28 – (number of “n/a” * 2); summary score = total sum/total possible sum.

### Outcomes

#### Quality of care (patient-level)

Ten studies reported patient-related outcomes such as health status, mortality and satisfaction (***Table 3***). Of the studies that compared primary care plus with usual specialist hospital care, most reported no significant differences in patient-related outcomes between the interventions [9,28,31]. One exception was the study of McCorkle et al. (2000) who reported that the risk of death was doubled in patients receiving usual specialist hospital care as compared to primary care plus (adjusted hazard ratio 2.04; confidence interval 1.33–3.12). Furthermore, a significant higher 2-year survival rate for a specific subgroup of patients (late stage cancer) was found in favour of the primary care plus intervention (67% versus 40%, p < 0.05). Quality of life did not significantly differ between the interventions [25].

**Table 3:** Outcomes of the included studies.


STUDY	PATIENT OUTCOMES (MORBIDITY, MORTALITY, HEALTH STATUS, QUALITY OF LIFE, PATIENT SATISFACTION, PATIENT COMPLIANCE, AND PATIENT SAFETY)	PROVIDER OUTCOMES (JOB WORKLOAD, JOB SATISFACTION, AND THE EXPERIENCES OF PAS/APNS)	COSTS AND COST-EFFECTIVENESS (INCLUDING UTILIZATION OF RESOURCES)	CARE OUTCOMES (HEALTH CARE ACTIVITIES/ROLES SUCH AS EXAMINATION, ADVICE, TREATMENTS; THE QUALITY OF THE HEALTH CARE; AND FACILITATORS AND BARRIERS)

Ansari et al. (2009)	**Health status** FEV_1_% pred. (intervention) baseline: 46.9 ± 19.8, follow-up: 48.1 ± 21.6; FEV_1_% pred. (comparison) baseline: 45.9 ± 19.0, follow-up: 53.5 ± 18.2. **Admission** 1/60 patients in the intervention group (UCT) required admission to hospital within 10 days.	–	–	

Bookbinder et al. (2011)	**Health status** N = 45 sign. reduction in physical symptom subscale score (z = –2/390, p = 0.003). **Admission** N = 27 (22%) referred from the intervention to hospice. N = 32 active cases transitioned to other services for continued care.	–	**Costs APN** 350 visits ($67,000 total yearly reimbursement), 140 first time visits ($238 per visit), 17 inpatient visits ($300 per visit), 193 follow-up visits ($102–170 per visit)	**Barriers** Obtaining services, reimbursement for NP, acquisition of new patients, geographic distribution of patients, no. of visits, medical management by the NP.

Gunn et al. (2000)	**Health status** Breastfeeding rate or amount, ns. **Patient satisfaction** Majority satisfied intervention (early discharge). **Admission** Re-admission to hospital: 6 wk.: 8.8% vs. 11.9%, p = 0.37; 6 mo.: 20.2% vs. 20.3%, p = 0.96.	–	–	

Jack et al. (2008)	**Patient compliance** Attendance rate intervention: >85% (usually <40%). Compliance with therapy was good.	–	–	–

Kemp, A.E. (2016)	**Admission** Referral to local health care resources: 0.03 (23; 3.5%) vs. 0.12 (105; 16.1%), p < .001). Referral to hospital: 0.02 (20; 3.2%) vs. 0.11 (91; 14%), p < .001). Ambulance transport to hospital rate: 0.01 (11; 1,7%) vs. 0.06 (47; 7,2%), p < .001).	–	**Costs** Estimated direct savings = £22,066.	

Lemelin, J. et al. (2007)	**Patient satisfaction** High levels of satisfaction = 88–100%. Preferred site = 63% at home, 37% hospital. **Adverse events** N = 0 adverse events or mortality. **Admission** N = 2 re-admitted to the inpatient service.	**Experiences physicians** Virtually all: at least satisfied with intervention. 88% = did not affect/affect in a positive way practice routine. **Experiences NP** All: very good quality of care. Majority: satisfied participation in decision-making. Some patients too ill for NP-profession, others not requiring NP-expertise.	–	**Facilitators** NP could act autonomously. **Barriers** No direct access to diagnostic tests and specialists, challenges in developing relationships, defining roles and establishing program ‘buy-in’ with medical staff.

Lucatorto et al. (2016)	**Health status** Hemoglobin A_1c_ and eGFR stages remained stable. **Medication** Angiotensin-convertin enzyme inhibitors/angiotensin receptor blockers: 20% increase; cholesterol-lowering medication: 27% increase; insulin: 10% increase; NSAIDs: 7% decrease.	**Experiences NP** Self-perceived confidence (diabetes) = 6.2; self-perceived confidence (renal disease) = 4.7; past experiences was related to higher confidence levels.	–	**Facilitators** Printed materials, collaboration, teamwork, experience, expertise, benchmarking, chance to network. **Barriers** Diverse patients, time to get lab. data, complexity of setting up shared medical appointment, patient transport issues.

Lukas et al. (2013)	**Admission** Sign. reduction in: no. of hospitalization (p = 0.000, *d* = 0.75), no. of days in hospital (p = 0.000, *d* = 0.81), 30-day readmission (p = 0.02, *OR* = 1.66). No sign. reduction in: emergency department visits (ns, *OR* = 1.20).	–	**Costs** Sign. reduction in: total costs for hospitalisation (p = 0.000, *d* = 0.52), variable costs for hospitalisation (p = 0.000, *d* = 0.53)	–

Maruthachalam et al. (2006)	**Patient satisfaction** 99% = satisfied clinic service (n = 447), 76% = had service on time (n = 342). Patients were willing to be investigated: less anxiety, better facilities, easier access. **Admission to flexible sigmoidoscopie** Median time in clinic = 35 days (range 1–180), in hospital = 87 days (range NR). **Admission to hospital after flexible sigmoidoscopy** 72% referral (n = 716), 28% no referral (n = 284).	–	**Costs** Flexible sigmoidoscopy in clinic = £270; flexible sigmoidoscopy in hospital = £396 (including equipment, salary, capital costs, costs for consumables)	–

McCorkle et al. (2000)	**Risk of death** Adjusted hazard ratio 2.4; CI 1.33–3.12; p =.001. **2-year survival rate** Early stage patients = 90.3% versus 87.6%, ns. Late stage patients = 67% versus 40%, p < 0.05. **Quality of life** No difference between intervention and usual care. **Admission** 32% intervention versus 27% usual care.	–	–	–

McLachlan et al. (2015)	**Medication** Sign. increase in aspirin use (p = 0.001), but not statin, angiotensin converter enzyme inhibitor, calcium channel blocker, beta blocker, thiazide and angiotensin receptor blocker (p>0.05). **Adverse events** 2% stroke (n = 9), 4% died (n = 18). **Admission/referral** 4% referred to cardiologist (n = 18), 1% redo valve surgery (n = 6), 1,5% required urgent admission (n = 7).	–	–	–

Moore, J.A. (2016)	**Admission** Intervention results in substantial reduction (–28%) in 30-day hospital readmission and emergency department visits.	–	–	**Facilitators** Daily interactions, tele monitoring, weekly interdisciplinary home health meetings, staff involvement, informative brochures. **Barriers** Lack of notification new patients, handwritten medical visit information, NP part-time availability, restrictive NP practice privileges.

Regan & Morgan (2015)	**Patient satisfaction** 1/8 patients stated: ‘This is an excellent idea. I feel a lot better in myself by being treated at home. I was in hospital for five wk., only needing one injection a day, and I was getting very frustrated.		–	**Barriers** Unpredictable, sporadic transfers to service, weekends and bank holidays, limited capacity and skills. **Facilitators** Multidisciplinary professional engagement, communication with medical lead, communication pathways, assessment, co-ordination and patient management.

Tozer & Lugton (2007)	–	**Experiences clinicians** Clinicians: in favor of the revised pathway. Nurses: convinced of its value. PCT team: has potential to become cost effective.	–	**Barriers** Distance travelled per visit varied (range 2–99 miles).

Whitaker et al. (2001)	**Effectiveness** No sign. differences in efficacy, duration and no. of treatments. Time between injection and reinjection was lower in intervention (1.5 wk.) than usual care (3.8 wk.). **Patient satisfaction** Home service was preferred over usual care (p = 0.001), efficacy improved (p = 0.001). **Adverse events** Similar in both groups except for sign. less dysphagia (p = 0.018) in the home group (7 versus 24 occasions). **Admission** N = 1 (intervention) was referred back	–	**Costs** Total cost per visit was not sign. different between the home injection group ($36.90/£23.36) and clinic group ($79.00/£50.01).	


FEV1 = Forced Expiratory Volume in the first second; NP = nurse practitioner.

Of the studies in which the implementation of the primary care plus intervention was evaluated, without a comparison with usual specialist hospital care, reported that the health status of patients either significantly improved from baseline (z = –2/390, p = 0.003) [21] or remained stable [29]. However, the use of some medications increased significantly, e.g. aspirin (p = 0.001) [26], angiotensin-convertin enzyme inhibitors/ – receptor blockers and cholesterol-lowering medication (20% and 27% increase, respectively) [29].

Patient satisfaction was reported in five studies. Most patients were highly satisfied with the primary care plus intervention in general (not compared with usual specialist hospital care) [8,24] Patients reported good facilities and easy access of primary care plus [24]. They were satisfied with the early discharge [9], being treated at home [27] and the prevention from being admitted to the hospital [28]. When compared to usual specialist hospital care, patients from one study preferred primary care plus over usual specialist hospital care [28]. Almost two-third of the patients from another study also preferred primary care plus over usual specialist hospital care. Patients who did not preferred primary care plus reported concerns about being left alone at home [8].

Eleven studies reported on admission or referral rates. Three studies which statistically compared rates between primary care plus and usual specialist hospital care either reported no significant differences between the interventions (20% versus 20% at 6 months follow-up, p = 0.96) [9] or reported a significant reduction in the number of admission or referral rates in favor of the primary care plus (3% versus 14%, p = <0.001 [20], and *d* = 0.75, p = 0.000 [23]). The significant reduction in the study of Kemp et al. (2016) was potentially a consequence of the ability of the nurse practitioners to close wounds and to prescribe.

Two studies that compared admission or referral rates between primary care plus and usual care or the national average reported either a comparable number of readmission hospital rates (32% versus 27% [25]), or a substantial reduction (–28%) in 30-day hospital readmission and emergency department visits in favor of the primary care plus [32]. A third study reported a slight increased number of referrals to “external medical consultants” such as orthopedic, neurologic/neurosurgical, wheelchair assessment, general medical, counseling, ENT and pain clinic (18/243 visits (7%; primary care plus) versus 13/210 visits (6%; usual specialist hospital care) [28]. They reported that the increased number of referrals seemed to be a consequence of the nurse practitioner having more time available during consultations and the ability to make a detailed appraisal of patients’ needs.

Studies reporting referral rates of primary care plus to the hospital (without comparing rates with usual specialist hospital care) reported rates ranging from 1,66%–72% [8,21,24,26,31]. Maruthachalam et al. (2006) furthermore reported that the median waiting time to the flexible sigmoidoscopy was more than halved when compared to the median waiting time to usual specialist hospital care prior to the implementation of primary care plus (35 versus 87 days). In addition, they reported that more capacity could be generated in the hospital by introducing primary care plus.

#### Quality of care (professional-level)

Three studies investigated health care professional experiences with primary care plus services [8,29,30]. These studies did not compare outcomes with usual specialist hospital care. All three studies reported positive experiences expressed by nurse practitioners. Nurse practitioners reported e.g. good quality of care, satisfaction with the extent to which they were involved in decision-making [8] and being convinced of the value of primary care plus [30]. Nurse practitioners in one study mentioned that self-perceived confidence levels were however not always optimal [29]. To overcome this, additional medical specialists were added to the team. Another study also reported that the nurse practitioner profession was not always the most appropriate as some patients seemed too complex while other patients did not require specialized nurse practitioner care [8]. An appropriate selection of professionals and patients is therefore of significant importance.

#### Costs

Four studies reported on costs related to primary care plus and usual specialist hospital care [20,23,24,28]. These studies reported lower total costs per visit in favour of primary care plus [24,28], reductions in total and variable costs for all hospitalizations in favour of primary care plus [23], and direct savings in total costs after implementing primary care plus [20]. Only two of these studies, however, statistically compared outcomes between the interventions. Whitaker et al. (2001) reported no significant difference in total cost per visit between the interventions, while Lukas et al. (2013) reported a significant reduction in total and variable costs for all hospitalizations in favour of the primary care plus intervention.

In addition, a fifth study reported costs of primary care plus but did not report nor compared this with usual specialist hospital care. Of notice is that this study needed to stop the primary care plus intervention after two years due to the fact there was lack of funding [21]. Overall, no results are reported about cost-effectiveness since no studies gathered information to perform a cost-effectiveness analysis.

#### Facilitators and barriers

Six studies reported facilitators and or barriers related to the implementation of primary care plus. General barriers were the difficulty to obtain equipment and to receive structural funding for primary care plus [8,21]. Some studies experienced difficulties in obtaining direct access to relevant patient information such as laboratory data or diagnostic tests [8,29,32]. In addition, the capacity of the nurse practitioner (e.g. part-time availability) as well as a part-time supply of patients negatively influenced a structural service in two studies [27,32]. Two studies from the USA furthermore reported that nurse practitioners were not permitted to act autonomously, and physician referral or prescription was needed [21,32]. In studies where nurse practitioners did have the permission to act autonomously, autonomy was experienced a facilitator [8]. Nurse practitioners in two studies reported to find it challenging to develop relationships with specialized staff or to set up a shared medical appointment [8,29]. However, as soon as collaboration with multiple caregivers seemed successful, this was deemed a facilitator and a strength of primary care plus service [27,29,32]. Support from specialized medical specialists such as having meetings to review patients and tele monitoring improved early detection of patients and improved nurse practitioners’ skills. Clear protocols about communication, assessment, co-ordination and management facilitated compliance of health care professionals and are therefore required for successful implementation of primary care plus provided by a nurse practitioner [29,32].

## Discussion

This systematic review aimed to gather international literature to get insight in the role of nurse practitioners and physician assistants in primary care plus and in the effectiveness, costs and influencing factors (barriers and facilitators) when implementing primary care plus with nurse practitioners or physician assistants. In summary, this systematic review included 15 studies in which primary care plus was provided by one or more nurse practitioners [8,9,20,21,22,23,24,25,26,27,28,29,30,31,32]. No studies involved physician assistants. Nurse practitioners mostly worked in a team of professionals and often received supervision from a medical specialist. The majority of the studies aimed at preventing referral to a hospital. A few on early discharge. Overall, the quality of care, both at patient-level and professional-level, appears to be guaranteed with possibly better access to healthcare and fewer referrals to the hospital. When implementing or investigating primary care plus delivered by nurse practitioners, facilitators to optimize success should be taken into account such as the ability to obtain equipment, direct access to patient information, structural funding, collaboration with health care professionals and the ability of the nurse practitioner to work autonomously. Since many studies had an observational or descriptive design, findings should be interpreted with caution.

Regarding its effectiveness, no difference between primary care plus provided by a nurse practitioner and usual specialist hospital care was reported in patient-related outcomes such as health status or quality of life. Our findings are in line with findings of a recently published systematic review in which primary care plus delivered by medical specialists was equally or more effective in nearly all studies in improving health status as compared to usual specialist hospital care [10]. Systematic reviews in which care delivered by nurse practitioners or physician assistants was compared with medical specialists showed that these professionals seem to be able to provide specialist care of equal effectivity [15,16,17]. This was reported in healthcare for the aging population [16], in primary care [17], in secondary care, acute internal medicine, emergency medicine, trauma and orthopaedics, and mental health [15]. Overall, this underlines that nurse practitioners and physician assistants can provide specialist care effectively, regardless of the setting (hospital, nursing home, primary care or primary care plus).

Our systematic review showed that patients satisfied the primary care plus intervention as it was easy accessible, patients could be treated at home and they were prevented from hospitalisation. This was reported by studies that were nearly all of at least adequate methodological quality. The findings are furthermore in accordance with findings of the systematic review of van Hoof et al. (2016) who reported high patient satisfaction as well [10]. Van Hoof et al. (2019) furthermore reported shorter waiting times and fewer follow-up visits in primary care plus. Our systematic review found either an equal number of hospital referrals/(re-)admissions or a significantly reduced number when compared to usual specialist hospital care. The reduced number could partly rely on the skills and the ability of the nurse practitioner to work autonomously. If the nurse practitioner can perform specialist care in primary care plus such as wound closure and medication prescriptions, no referral to the hospital is needed. A reduction in the number of hospital referrals might in turn reduce waiting times in hospitals.

Only two studies (of high methodological quality) statistically compared costs between the interventions, showing varying results. This is in line with previous performed systematic reviews on substituting physician assistants and nurse practitioners with medical specialists [15,16,17] and a systematic review on shifting specialist care to primary care by medical specialists [10]. The varying results may be a consequence of the different indicators which were taken into account by calculating costs. For example, one study included in this systematic review calculated costs per visit [28], while another study calculated costs for hospitalisation (e.g. prescription, referral, salary of providers etcetera) [23]. The latter relates more to the number of patients that have been referred or treated in the hospital, while the former relates more to the direct cost of the provided health care itself. To be able to compare costs between interventions, as well as cost outcomes between studies, it should be recommended to analyse costs from a societal and health care sector perspective [33]. Such outcomes in turn, can be used for a cost-effectiveness analysis to determine which intervention should be provided. In this systematic review, no studies performed a cost-effectiveness analysis. Therefore, no conclusions can be drawn at this field.

### Methodological Considerations

A strength of our systematic review was the help of an experienced information specialist in conducting the search. The enhanced search, in combination with a reference check at the end of the procedure, reduced the risk of missing relevant articles. Another strength of the study was the fact multiple researchers were involved in the selection of articles, the risk of bias assessment and the data extraction. Involving multiple researchers reduces the risk of selection bias, inadequate risk of bias assessment, and incomplete data extraction. Defining primary care plus was, however, challenging. This was due to the fact health care is organized differently in each country. The Dutch health care system is divided into primary care (e.g. family practice) and secondary care (hospital care), and therefore primary care plus can be defined. During the selection procedure, review authors critically appraised whether specialist care was shifted from the hospital setting and integrated to primary care or home care setting. As described in the methods section, review authors discussed interpretation of eligibility criteria of the first 30 records (sorted on best matches) at the start of the selection procedure. Although this optimized the selection procedure, there is still a possibility that relevant studies have been interpreted wrongly and therefore have not been included in this systematic review. Furthermore, we excluded studies investigating solely newly developed “additional” care. Since primary care plus was not always completely identical to usual specialist hospital care and occasionally supplemented with additional care (e.g. McCorkle et al. (2000)), this might have caused heterogeneity of the results of primary care plus. Another limitation is that most studies did not report characteristics of the nurse practitioner in detail. Therefore, it is not possible to rule out that all nurse practitioners in the included studies obtained a master’s degree (QLF 7). Most studies furthermore investigated care provided by one professional only (n = 1). It may be questioned whether the studies investigated the effectiveness of the intervention or the performance of the individual professional. Finally, no studies were included investigating physician assistants in primary care. An explanation could be that the physician assistant profession in many counties is relatively new. Despite this, we hope health care professionals and researchers will set up and conduct studies about potentials roles of physician assistants in future. Physician assistants in many countries can have similar roles and rights as nurse practitioners [13,14], and could therefore be of value in primary care plus.

As became clear in this systematic review, primary care plus provided by nurse practitioners is investigated in only a few studies yet and with restricted methodological quality. Most studies used a descriptive design and reported selection procedures, population characteristics and results in insufficient detail. For future, it is recommended to perform cost-effectiveness studies comparing a team of nurse practitioners in primary care plus with usual care in hospitals. Such studies are needed to draw firm conclusions about the potential of nurse practitioners as well as physician assistants in primary care plus.

## Conclusions

This systematic review shows that primary care plus, an elaboration of integrated care models, provided by a nurse practitioner is still in its infancy, but seems a potential opportunity for well-defined patient populations. The quality of care, both at patient-level and professional-level, appears to be guaranteed with possibly better access to healthcare and fewer referrals to the hospital. Since most studies had an observational or descriptive design, and the methodological quality was restricted, findings should be interpreted with caution. No studies were found reporting on physician assistants in primary care plus. More practices with physician assistants and nurse practitioners in primary care plus should be implemented and evaluated systematically, including a cost-effectiveness analysis. This systematic review will help policy makers and professionals to discuss about shifting specialist care from hospitals to primary or community care, at or close to patients’ home and within this the potential role of physician assistants and nurse practitioners.

## Additional File

The additional file for this article can be found as follows:

10.5334/ijic.5485.s1 Appendix 1. Search strategy.

## References

[1] World Health Organization. Regional Office for Europe. Healthy ageing. http://www.euro.who.int/en/health-topics/Life-stages/healthy-ageing/healthy-ageing. Accessed 23 May 2019.

[2] van Oostrom SH, Picavet HSJ, van Gelder BM, Lemmens LC, Hoeymans N, Verheij RA, Schellevis FG and Baan CA. Multimorbiditeit en comorbiditeit in de Nederlandse bevolking – gegevens van huisartsenpraktijken [Multimorbidity and comorbidity in the Dutch population – data from general practice]. Ned Tijdschr Geneeskd. 2011; 155: A3193. 21586184

[3] Peiro S and Maynard A. Variations in health care delivery within the European Union. Eur J Public Health. 2015; 25(1): 1–2. DOI: 10.1093/eurpub/cku223 25690122

[4] Baxter S, Johnson M, Chambers D, Sutton A, Goyder E and Booth A. The effects of integrated care: a systematic review of UK and international evidence. BMC Health Serv Res. 2018; 10; 18(1): 350 DOI: 10.1186/s12913-018-3161-3 29747651 PMC5946491

[5] van Hoof SJ, Kroese ME, Spreeuwenberg MD, Elissen AM, Meerlo RJ, Hanraets MM and Ruwaard D. Substitution of Hospital Care with Primary Care: Defining the Conditions of Primary Care Plus. Int J Integr Care. 2016; 14; 16(1): 12 DOI: 10.5334/ijic.2446 PMC5015530 27616956

[6] Giannitrapani KF, Leung L, Huynh AK, Stockdale SE, Rose D, Needleman J, Yano EM, Meredith L and Rubenstein LV. Interprofessional training and team function in patient-centred medical home: Findings from a mixed method study of interdisciplinary provider perspectives. Journal of Interprofessional Care 2018; 32(6): 735–744, DOI: 10.1080/13561820.2018.1509844 30156933

[7] Quanjel TCC, Spreeuwenberg MD, Struijs JN, Baan CA and Ruwaard D. Substituting hospital-based outpatient cardiology care: The impact on quality, health and costs. PLoS ONE. 2019; 14(5): e0217923 DOI: 10.1371/journal.pone.0217923 31150520 PMC6544378

[8] Lemelin J, Hogg WE, Dahrouge S, Armstrong CD, Martin CM, Zhang W, Dusseault JA, Parsons-Nicota J, Saginur R and Viner G. Patient, informal caregiver and care provider acceptance of a hospital in the home program in Ontario, Canada. BMC Health Serv Res. 2007; 17; 7: 130 DOI: 10.1186/1472-6963-7-130 17705866 PMC2020484

[9] Gunn TR, Thompson JM, Jackson H, McKnight S, Buckthought G and Gunn AJ. Does early hospital discharge with home support of families with preterm infants affect breastfeeding success? A randomized trial. Acta Paediatr. 2000; 89(11): 1358–63. DOI: 10.1080/080352500300002570 11106050

[10] van Hoof SJM, Quanjel TCC, Kroese MEAL, Spreeuwenberg MD and Ruwaard D. Substitution of outpatient hospital care with specialist care in the primary care setting: A systematic review on quality of care, health and costs. PLoS One. 2019; 1; 14(8): e0219957 DOI: 10.1371/journal.pone.0219957 31369567 PMC6675042

[11] Lambregts J. Projectgroep V&V 2020, Grotendorst A. Beroepsprofiel verpleegkundig specialist [Professional profile nurse specialist]. Verpleegkundigen & Verzorgenden 2020 2012; 4, 1–53.

[12] Freund T, Everett C, Griffiths P, Hudon C, Naccarella L and Laurant M. Skill mix, roles and remuneration in the primary care workforce: who are the healthcare professionals in the primary care teams across the world? Int J Nurs Stud. 2015; 52(3): 727–43. DOI: 10.1016/j.ijnurstu.2014.11.014. 25577306

[13] Ministerie van Volksgezondheid, Welzijn en Sport (ed.) [Ministry of Health, Welfare and Sport]. Besluit opleidingseisen en deskundigheidsgebied physician assistant [Decree on training requirements and expertise area physician assistant] Den Haag; 2018.

[14] De Bruijn-Geraets DP, van Eijk-Hustings YJL, Bessems-Beks MCM, Essers BAB, Dirksen CD and Vrijhoef HJM. National mixed methods evaluation of the effects of removing legal barriers to full practice authority of Dutch nurse practitioners and physician assistants. BMJ Open. 2018; 22; 8(6): e019962 DOI: 10.1136/bmjopen-2017-019962 PMC6020970 29934382

[15] Halter M, Wheeler C, Pelone F, Gage H, de Lusignan S, Parle J, Grant R, Gabe J, Nice L and Drennan VM. Contribution of physician assistants/associates to secondary care: a systematic review. BMJ Open 2018; 8(6): e019573 DOI: 10.1136/bmjopen-2017-019573 PMC6020983 29921680

[16] Lovink MH, Persoon A, Koopmans RTCM, Van Vught AJAH, Schoonhoven L and Laurant MGH. Effects of substituting nurse practitioners, physician assistants or nurses for physicians concerning healthcare for the ageing population: a systematic literature review. J Adv Nurs. 2017; 73(9): 2084–2102. DOI: 10.1111/jan.13299 28299815

[17] Laurant M, Reeves D, Hermens R, Braspenning J, Grol R and Sibbald B. Substitution of doctors by nurses in primary care. Cochrane Database Syst Rev. 2005 4 18; (2): CD001271 DOI: 10.1002/14651858.CD001271.pub2 15846614

[18] Ouzzani M, Hammady H, Fedorowicz Z and Elmagarmid A. Rayyan – a web and mobile app for systematic reviews. Systematic Reviews (2016) 5: 210 DOI: 10.1186/s13643-016-0384-4 27919275 PMC5139140

[19] Kmet L, Lee R and Cook L. Standard Quality Assessment Criteria for Evaluating Primary Research Papers From a Variety of Fields Alberta: Alberta Heritage Foundation for Medical Research; 2014.

[20] Kemp AE. Mass-gathering Events: The Role of Advanced Nurse Practitioners in Reducing Referrals to Local Health Care Agencies. Prehosp Disaster Med. 2016; 31(1): 58–63. DOI: 10.1017/S1049023X15005543 26732288

[21] Bookbinder M, Glajchen M, McHugh M, Higgins P, Budis J, Solomon N, Homel P, Cassin C and Portenoy RK. Nurse practitioner-based models of specialist palliative care at home: sustainability and evaluation of feasibility. J Pain Symptom Manage. 2011; 41(1): 25–34. DOI: 10.1016/j.jpainsymman.2010.04.011 20851569

[22] Jack K, Willott S, Manners J, Varnam MA and Thomson BJ. Clinical trial: a primary-care-based model for the delivery of anti-viral treatment to injecting drug users infected with hepatitis C. Aliment Pharmacol Ther. 2009; 29(1): 38–45. DOI: 10.1111/j.1365-2036.2008.03872.x 18945252

[23] Lukas L, Foltz C and Paxton H. Hospital outcomes for a home-based palliative medicine consulting service. J Palliat Med. 2013; 16(2): 179–84. DOI: 10.1089/jpm.2012.0414 23308377

[24] Maruthachalam K, Stoker E, Nicholson G and Horgan AF. Nurse led flexible sigmoidoscopy in primary care – the first thousand patients. Colorectal Dis. 2006; 8(7): 557–62. DOI: 10.1111/j.1463-1318.2006.00973.x 16919106

[25] McCorkle R, Strumpf NE, Nuamah IF, Adler DC, Cooley ME, Jepson C, Lusk EJ and Torosian M. A specialized home care intervention improves survival among older post-surgical cancer patients. J Am Geriatr Soc. 2000; 48(12): 1707–13. DOI: 10.1111/j.1532-5415.2000.tb03886.x 11129765

[26] McLachlan A, Sutton T, Ding P and Kerr A. A Nurse Practitioner Clinic: A Novel Approach to Supporting Patients Following Heart Valve Surgery. Heart Lung Circ. 2015 11; 24(11): 1126–33. DOI: 10.1016/j.hlc.2015.04.064 25991391

[27] Regan K and Morgan J. Implementing a nurse-led community intravenous antibiotic service. Primary Health Care. 2015; 25(7) 18–24.

[28] Whitaker J, Butler A, Semlyen JK and Barnes MP. Botulinum toxin for people with dystonia treated by an outreach nurse practitioner: a comparative study between a home and a clinic treatment service. Arch Phys Med Rehabil. 2001; 82(4): 480–4. DOI: 10.1053/apmr.2001.21843 11295008

[29] Lucatorto MA, Watts SA, Kresevic D, Burant CJ and Carney KJ. Impacting the Trajectory of Chronic Kidney Disease With ARPN-Led Renal Teams. Nurs Adm Q. 2016; 40(1): 76–86. DOI: 10.1097/NAQ.0000000000000148 26636237

[30] Tozer D and Lugton C. Cancer genetics in rural primary care: a pilot nurse-led service using a new mobile IT system. Fam Cancer. 2007; 6(2): 221–9. DOI: 10.1007/s10689-007-9133-0 17520350

[31] Ansari K, Shamssain M, Farrow M and Keaney NP. Hospital-at-home care for exacerbations of chronic obstructive pulmonary disease: an observational cohort study of patients managed in hospital or by nurse practitioners in the community. Chron Respir Dis. 2009; 6(2): 69–74. DOI: 10.1177/1479972309102728 19411566

[32] Moore JM. Evaluation of the efficacy of a nurse practitioner-led home-based congestive heart failure clinical pathway. Home Health Care Services Quarterly, 2016; 35(1): 39–51, DOI: 10.1080/01621424.2016.1175992 27064361

[33] Sanders GD, Neumann PJ, Basu A, Brock DW, Feeny D, Krahn M, Kuntz KM, Meltzer DO, Owens DK, Prosser LA, Salomon JA, Sculpher MJ, Trikalinos TA, Russell LB, Siegel JE and Ganiats TG. Recommendationsy for conduct, methodological practices, and reporting of cost-effectiveness analyses: Second Panel on Cost Effectiveness in Health and Medicine. JAMA 2016; 316(10): 1093–103. DOI: 10.1001/jama.2016.12195 27623463

