# Supplementary material for: Physician Assistants and Nurse Practitioners in Primary Care Plus: A Systematic Review
Source: Int J Integr Care. 2021 Feb 12;21(1):6. doi: 10.5334/ijic.5485 (PMC7879997; doi:10.5334/ijic.5485)
Supplement: Appendix 1. — Search strategy. [file ijic-21-1-5485-s1.pdf]

APPENDIX 1. SEARCH STRATEGY

CINAHL (EBSCO)

S1 (MH "Physician Assistants") OR (MH "Clinical Nurse Specialists") OR (MH "Advanced Practice Nurses") OR (MH "Nurse Practitioners+") OR (MH "Advanced Nursing Practice") OR (TI ((physician* N1 (assistant* OR associate* OR extender* OR substitute*)) OR (advance* W1 (provider* OR nurs * OR practice*)) OR ((((non OR none) W1 physician*) OR nonphysician* OR Midlevel OR (mid W1 level)) W2 (clinic* OR professional* OR provider* OR worker* OR personnel OR practitioner* OR staff OR specialist* OR (first W1 assistant*))) OR ((Clinical OR doctor*) W1 assistant*) OR ((nurs* OR Specialist OR emergency) W1 practitioner*) OR (Nurse N1 (clinician* OR specialist*)) OR (Academic N1 nurs*) OR APN OR APNS OR Feldsher)) OR (AB ((physician* N1 (assistant* OR associate* OR extender* OR substitute*)) OR (advance* W1 (provider* OR nurs * OR practice*)) OR ((((non OR none) W1 physician*) OR nonphysician* OR Midlevel OR (mid W1 level)) W2 (clinic* OR professional* OR provider* OR worker* OR personnel OR practitioner* OR staff OR specialist* OR (first W1 assistant*))) OR ((Clinical OR doctor*) W1 assistant*) OR ((nurs* OR Specialist OR emergency) W1 practitioner*) OR (Nurse N1 (clinician* OR specialist*)) OR (Academic N1 nurs*) OR APN OR APNS OR Feldsher)) OR (SU ((physician* N1 (assistant* OR associate* OR extender* OR substitute*)) OR (advance* W1 (provider* OR nurs * OR practice*)) OR ((((non OR none) W1 physician*) OR nonphysician* OR Midlevel OR (mid W1 level)) W2 (clinic* OR professional* OR provider* OR worker* OR personnel OR practitioner* OR staff OR specialist* OR (first W1 assistant*))) OR ((Clinical OR doctor*) W1 assistant*) OR ((nurs* OR Specialist OR emergency) W1 practitioner*) OR (Nurse N1 (clinician* OR specialist*)) OR (Academic N1 nurs*) OR APN OR APNS OR Feldsher*))

S2 (MH "Home Health Care+") OR (MH "Health Care Delivery, Integrated") OR (MH "Health Care Reform") OR (MH "Hospital-Physician Joint Ventures") OR (MH "Shared Services, Health Care") OR (TI (((Transmural OR Integrat*) N2 (care OR health* OR service*)) OR (((Co W1 located) OR Shifting OR Substitution) N1 care) OR (consultation N1 liaison*) OR (Nurs* N1 led*) OR Nurseled* OR (Hospital N1 Based N1 Home N1 Car*) OR (relocation N2 (service* OR care)) OR ((care OR health*) N1 Reform*))) OR (AB (((Transmural OR Integrat*) N2 (care OR health* OR service*)) OR (((Co W1 located) OR Shifting OR Substitution) N1 care) OR (consultation N1 liaison*) OR (Nurs* N1 led*) OR Nurseled* OR (Hospital N1 Based N1 Home N1 Car*) OR (relocation N2 (service* OR care)) OR ((care OR health*) N1 Reform*))) OR (SU (((Transmural OR Integrat*) N2 (care OR health* OR service*)) OR (((Co W1 located) OR Shifting OR Substitution) N1 care) OR (consultation N1 liaison*) OR (Nurs* N1 led*) OR Nurseled* OR (Hospital N1 Based N1 Home N1 Car*) OR (relocation N2 (service* OR care)) OR ((care OR health*) N1 Reform*)))

S3 MH "Primary Health Care" OR (TI (primary N1 (care OR health*))) OR (AB (primary N1 (care OR health*))) OR (SU (primary N1 (care OR health*)))

S4 MH "Hospitals+" OR MH "Secondary Health Care" OR (TI (hospital OR hospitals OR (secondary N1 (care OR health*)))) OR (AB (hospital OR hospitals OR (secondary N1 (care OR health*)))) OR (SU (hospital OR hospitals OR (secondary N1 (care OR health*))))

S5 S3 AND S4

S6 S2 OR S5

S7 S1 AND S6

Cochrane Database of Systematic Reviews

#1 ((physician* NEAR/1 (assistant* OR associate* OR extender* OR substitute*)) OR (advance* NEAR/1 (provider* OR nurs * OR practice*)) OR ((((non OR none) NEAR/1 physician*) OR nonphysician* OR Midlevel OR (mid NEAR/1 level)) NEAR/2 (clinic* OR professional* OR provider* OR worker* OR personnel OR practitioner* OR staff OR specialist* OR (first NEAR/1 assistant*))) OR ((Clinical OR doctor*) NEAR/1 assistant*) OR ((nurs* OR Specialist OR emergency) NEAR/1 practitioner*) OR (Nurse NEAR/1 (clinician* OR specialist*)) OR (Academic NEAR/1 nurs*) OR APN OR APNS OR Feldsher):ti,ab,kw

#2 (((Transmural OR Integrat*) NEAR/2 (care OR health* OR service*)) OR (((Co NEAR/1 located) OR Shifting OR Substitution) NEAR/1 care) OR (consultation NEAR/1 liaison*) OR (Nurs* NEAR/1 led*) OR Nurseled* OR (Hospital NEAR/1 Based NEAR/1 Home NEAR/1 Car*) OR (relocation N2 (service* OR care)) OR ((care OR health*) NEAR/1 Reform*)):ti,ab,kw

#3 (primary NEAR/1 (care OR health*)):ti,ab,kw
#4 (hospital OR hospitals OR (secondary NEAR/1 (care OR health*))):ti,ab,kw
#5 #3 AND #4
#6 #2 OR #5
#7 #1 AND #6

Embase (Ovid)

1 physician assistant/ OR nurse specialist/ or advanced practice nurse/ or clinical nurse specialist/ OR exp nurse practitioner/ OR advanced practice nursing/ OR ((physician* ADJ1 (assistant* OR associate* OR extender* OR substitute*)) OR (advance* ADJ1 (provider* OR nurs* OR practice*)) OR ((((non OR none) ADJ1 physician*) OR nonphysician* OR Midlevel OR (mid ADJ1 level)) ADJ2 (clinic* OR professional* OR provider* OR worker* OR personnel OR practitioner* OR staff OR specialist* OR (first ADJ1 assistant*))) OR ((Clinical OR doctor*) ADJ1 assistant*) OR ((nurs* OR Specialist OR emergency) ADJ1 practitioner*) OR (Nurse ADJ1 (clinician* OR specialist*)) OR (Academic ADJ1 nurs*) OR APN OR APNS OR Feldsher).ti,ab,kw.
2 exp home care/ OR integrated health care system/ OR (((Transmural OR Integrat*) ADJ2 (care OR health* OR service*)) OR (((Co ADJ1 located) OR Shifting OR Substitution) ADJ1 care) OR (consultation ADJ1 liaison*) OR (Nurs* ADJ1 led*) OR Nurseled* OR (Hospital ADJ1 Based ADJ1 Home ADJ1 Car*) OR (relocation ADJ2 (service* OR care)) OR ((care OR health*) ADJ1 Reform*)).ti,ab,kw.
3 primary medical care/ OR (primary ADJ1 (care OR health*)).ti,ab,kw.
4 exp secondary health care/ OR (hospital OR hospitals OR (secondary ADJ1 (care OR health*))).ti,ab,kw.
5 3 AND 4
6 2 OR 5
7 1 AND 6
8 limit 7 to conference abstract status
9 7 NOT 8
10 limit 9 to yr="1990 -Current"

PubMed

#1 “Physician Assistants"[mesh] OR physician assistant*[tiab] OR physician associate*[tiab] OR physician extender*[tiab] OR physicians assistant*[tiab] OR physicians extender*[tiab] OR physician substitute*[tiab] OR “nurse practitioners”[mesh] OR "Advanced Practice Nursing”[mesh] OR advance nurs*[tiab] OR advanced nurs*[tiab] OR nurse practitioner*[tiab] OR "Nurse Specialists"[Mesh] OR Nurse clinician*[tiab] OR Nurse specialist*[tiab] OR Academic nurs*[tiab] OR APN[tiab] OR APNS[tiab] OR APN S[tiab] OR specialist nurs*[tiab] OR Assistant physician*[tiab] OR advance practi*[tiab] OR advanced practi*[tiab] OR advanced providers[tiab] OR emergency practitioner*[tiab] OR feldsher*[tiab] OR mid level clinicians[tiab] OR mid level health care professionals[tiab] OR mid level health care provider*[tiab] OR mid level health care workers[tiab] OR mid level health professionals[tiab] OR mid level health providers[tiab] OR mid level health workers[tiab] OR mid level healthcare workers[tiab] OR mid level medical workers[tiab] OR mid level personnel[tiab] OR mid level practitioners[tiab] OR mid level professionals[tiab] OR mid level provider[tiab] OR mid level staff[tiab] OR mid level workers[tiab] OR midlevel clinician*[tiab] OR midlevel health care professional*[tiab] OR midlevel health care provider*[tiab] OR midlevel health care[tiab] OR midlevel health provider*[tiab] OR midlevel health worker*[tiab] OR midlevel personnel[tiab] OR midlevel practitioner*[tiab] OR midlevel professional*[tiab] OR midlevel provider*[tiab] OR non physician clinic staff[tiab] OR non physician clinicians[tiab] OR non physician first assistants[tiab] OR non physician health care personnel[tiab] OR non physician health care professionals[tiab] OR non physician health care providers[tiab] OR non physician health care workers[tiab] OR non physician health professionals[tiab] OR non physician health providers[tiab] OR non physician health workers[tiab] OR non physician healthcare professionals[tiab] OR non physician healthcare providers[tiab] OR non physician healthcare workers[tiab] OR non physician medical personnel[tiab] OR non physician personnel[tiab] OR non physician practice staff[tiab] OR non physician primary care providers[tiab] OR non physician professionals[tiab] OR non physician provider[tiab] OR non

physician providers[tiab] OR nonphysician clinic*[tiab] OR nonphysician medical personnel[tiab] OR nonphysician personnel[tiab] OR nonphysician practitioner*[tiab] OR nonphysician primary care clinicians[tiab] OR nonphysician primary care providers[tiab] OR nonphysician specialists[tiab] OR nonphysician staff[tiab] OR Clinical assistant*[tiab] OR Doctor s assistant*[tiab] OR Doctors assistant*[tiab] OR Specialist practitioner*[tiab]

#2 "Home Care Services"[Mesh:NoExp] OR "Home Care Services, Hospital-Based"[Mesh] OR "Delivery of Health Care, Integrated"[Mesh] OR "Hospital-Physician Joint Ventures"[Mesh:NoExp] OR Integrated care[tiab] OR Integrated health*[tiab] OR Substitution of care[tiab] OR Shifting care[tiab] OR Co-located care[tiab] OR consultation liaison*[tiab] OR transmural care[tiab] OR transmural health care[tiab] OR integrated service*[tiab] OR “Int J Integr Care”[ta] OR care integration*[tiab] OR integrated comprehensive car*[tiab] OR Integrated Managed Car*[tiab] OR integrated primary care[tiab] OR Nurse led*[tiab] OR Nursing led*[tiab] OR Nurseled*[tiab] OR Care substit* OR Hospital Based Home Car*[tiab] OR relocation of service*[tiab] OR relocation of care*[tiab] OR care relocation*[tiab] OR services relocation*[tiab] OR "Health Care Reform"[Mesh] OR Healthcare Reform*[tiab] OR care Reform*[tiab] OR health reform*[tiab]

#3 "Primary Health Care"[Mesh:NoExp] OR primary care[tiab] OR primary health*[tiab]

#4 "Hospitals"[Mesh] OR "Secondary Care"[Mesh] OR hospital[tiab] OR hospitals[tiab] OR secondary care[tiab]

#5 #3 AND #4

#6 #2 OR #5

#7 #1 AND #6

Web of Science

#1 TS=((physician* NEAR/1 (assistant* OR associate* OR extender* OR substitute*)) OR (advance* NEAR/1 (provider* OR nurs * OR practice*)) OR ((((“non” OR “none”) NEAR/1 physician*) OR nonphysician* OR Midlevel* OR (“mid” NEAR/1 “level”)) NEAR/2 (clinic* OR professional* OR provider* OR worker* OR “personnel” OR practitioner* OR “staff” OR specialist* OR (“first” NEAR/1 assistant*))) OR ((“Clinical” OR doctor*) NEAR/1 assistant*) OR ((nurs* OR “Specialist” OR “emergency”) NEAR/1 practitioner*) OR (“Nurse” NEAR/1 (clinician* OR specialist*)) OR (“Academic” NEAR/1 nurs*) OR “APN” OR “APNS” OR Feldsher*)

#2 TS=(((“Transmural” OR Integrat*) NEAR/2 (“care” OR health* OR service*)) OR (((“Co” NEAR/1 “located”) OR “Shifting” OR “Substitution”) NEAR/1 “care”) OR (“consultation” NEAR/1 liaison*) OR (Nurs* NEAR/1 led*) OR Nurseled* OR (“Hospital” NEAR/1 “Based” NEAR/1 “Home” NEAR/1 Car*) OR (“relocation” N2 (service* OR “care”)) OR ((“care” OR health*) NEAR/1 Reform*))

#3 TS=(“primary” NEAR/1 (“care” OR health*))

#4 TS=(“hospital” OR “hospitals” OR (“secondary” NEAR/1 (“care” OR health*)))

#5 #3 AND #4

#6 #2 OR #5

#7 #1 AND
